# Longitudinal associations of screen time, physical activity, and sleep duration with body mass index in U.S. youth

**DOI:** 10.1186/s12966-024-01587-6

**Published:** 2024-04-02

**Authors:** Jennifer Zink, Robert Booker, Dana L. Wolff-Hughes, Norrina B. Allen, Mercedes R. Carnethon, Shaina J. Alexandria, David Berrigan

**Affiliations:** 1 Division of Cancer Control and Population Sciences, Behavioral Research Program, Health Behaviors Research Branch, National Cancer Institute, 9609 Medical Center Drive, Rockville, MD 20850 USA; 2 Department of Preventive Medicine, Feinberg School of Medicine, Northwestern University, 680 N Lake Shore Drive, Chicago, IL 60611 USA; 3 Division of Cancer Control and Population Sciences, Epidemiology and Genomics Research Program, Risk Factors Assessment Branch, National Cancer Institute, 9609 Medical Center Drive, Rockville, MD 20850 USA

**Keywords:** ABCD study, Movement behaviors, Obesity, Youth

## Abstract

**Background:**

Youth use different forms of screen time (e.g., streaming, gaming) that may be related to body mass index (BMI). Screen time is non-independent from other behaviors, including physical activity and sleep duration. Statistical approaches such as isotemporal substitution or compositional data analysis (CoDA) can model associations between these non-independent behaviors and health outcomes. Few studies have examined different types of screen time, physical activity, and sleep duration simultaneously in relation to BMI.

**Methods:**

Data were baseline (2017–2018) and one-year follow-up (2018–2019) from the Adolescent Brain Cognitive Development Study, a multi-site study of a nationally representative sample of U.S. youth (*N* = 10,544, mean [SE] baseline age = 9.9 [0.03] years, 48.9% female, 45.4% non-White). Participants reported daily minutes of screen time (streaming, gaming, socializing), physical activity, and sleep. Sex-stratified models estimated the association between baseline behaviors and follow-up BMI *z*-score, controlling for demographic characteristics, internalizing symptoms, and BMI *z*-score at baseline.

**Results:**

In females, isotemporal substitution models estimated that replacing 30 min of socializing (β [95% CI] = -0.03 [-0.05, -0.002]), streaming (-0.03 [-0.05, -0.01]), or gaming (-0.03 [-0.06, -0.01]) with 30 min of physical activity was associated with a lower follow-up BMI *z*-score. In males, replacing 30 min of socializing (-0.03 [-0.05, -0.01]), streaming (-0.02 [-0.03, -0.01]), or gaming (-0.02 [-0.03, -0.01]) with 30 min of sleep was associated with a lower follow-up BMI *z*-score. In males, replacing 30 min of socializing with 30 min of gaming was associated with a lower follow-up BMI *z*-score (-0.01 [-0.03, -0.0001]). CoDA estimated that in males, a greater proportion of time spent in baseline socializing, relative to the remaining behaviors, was associated with a higher follow-up BMI *z*-score (0.05 [0.02, 0.08]). In females, no associations between screen time and BMI were observed using CoDA.

**Conclusions:**

One-year longitudinal associations between screen time and BMI may depend on form of screen time, what behavior it replaces (physical activity or sleep), and participant sex. The alternative statistical approaches yielded somewhat different results. Experimental manipulation of screen time and investigation of biopsychosocial mechanisms underlying the observed sex differences will allow for causal inference and can inform interventions.

**Supplementary Information:**

The online version contains supplementary material available at 10.1186/s12966-024-01587-6.

## Background

An estimated 20% of youth in the United States (U.S.) have obesity as measured by body mass index (BMI; kg⋅m^−2^) [1]. High BMI early in life can persist or increase into adulthood [2], indicating that obesity during childhood may be linked to risk for cardiometabolic disease and several types of cancer later in life [3–5]. Therefore, promoting a healthy BMI in youth is a promising prevention approach for obesity-related morbidity across the life course [6, 7].

The association between BMI and chronic disease has sparked interest in understanding highly prevalent and modifiable behavioral risk factors to inform strategies for promoting healthy BMI in youth. Prior to the COVID-19 pandemic, it was estimated that youth in the U.S. spent an average of 4 h per day on screen time, such as gaming, streaming, and socializing [8]. In a recent study, these forms of screen time were longitudinally associated with higher BMI [9]. However, streaming, gaming, and socializing can be correlated with each other, and their independent associations with BMI remain under-explored. Further, there are sex differences in screen time preferences in youth. Males tend to spend more time on gaming, while females spend more time on socializing [10]; given the potential for each form of screen time to differentially relate to BMI [11], hand-in-hand with the emergence of sex differences in body composition during the child-to-adolescent transition [12], exploring the association between different forms of screen time and BMI by sex is warranted.

In addition, screen time is correlated with other behaviors that are important for weight regulation, including physical activity and sleep duration [13, 14]. Due to the finite amount of time within a day, the displacement hypothesis postulates that screen time can displace opportunities to be physically active and sleep [15, 16], highlighting one potential mechanism linking screen time to BMI. Yet, few studies of screen time and BMI account for physical activity and sleep duration, making it difficult to determine the relative contributions of each of these behaviors to overweight/obesity risk.

Two common statistical methods to model the association between screen time, physical activity, and sleep duration simultaneously with BMI are isotemporal substitution and compositional data analysis (CoDA). Isotemporal substitution is a method to estimate the association between hypothetically replacing one behavior for an equal amount of time of another behavior and health outcomes [17]. These models recognize that the association between a given behavior and an outcome may be dependent upon what behavior it displaces [17]. A longitudinal isotemporal substitution analysis among ~ 700 children found that a replacement of 30 min of screen time with 30 min of physical activity was related to a lower BMI two years later [18]. However, these replacement associations were not observed between screen time and sleep, perhaps because the sample achieved adequate sleep on average (~ 9 h per night) [18]. CoDA examines the association between co-dependent behaviors (e.g., screen time, physical activity, sleep) and health [19]. As time is finite, this approach treats behaviors as proportions of the day and therefore estimates the association between the proportion of time spent in one behavior relative to the proportion of time spent in the remaining behaviors and health outcomes [19]. Isotemporal substitution and CoDA are both regression models, differing in that untransformed data are used in isotemporal substitution models, while isometric logarithmic ratio transformed data are used in CoDA [17, 19]. By using these two statistical approaches, we can begin to understand the combined and relative associations of screen time, physical activity, and sleep duration with BMI in youth.

Taken together, we examined whether different forms of screen time were associated with BMI, while also accounting for physical activity and sleep duration using sex-stratified isotemporal substitution analysis and CoDA in a one-year longitudinal study of participants from the Adolescent Brain Cognitive Development (ABCD) Study. We hypothesized that replacing screen time with an equal amount of time in physical activity or sleep (via isotemporal substitution analysis) would be associated with a lower BMI one year later. Similarly with CoDA, we hypothesized that a greater proportion of time spent on screens, relative time spent in the remaining behaviors, would be associated with a higher BMI one year later. Lastly, we hypothesized that these associations would depend on form of screen time and participant sex. The present study can demonstrate the importance of collectively examining different forms of screen time, physical activity, and sleep duration in relation to BMI in youth; and more broadly, can inform future research that uses similar approaches to understand how these behaviors jointly relate to physical and mental health across populations.

## Methods

### Study sample

Data were from the ABCD Study, a multi-site longitudinal study coordinated by the U.S. National Institutes of Health [20]. Participants (*N* = 11,876) aged 9 to 11 years old at baseline were recruited via a school-based strategy designed to obtain a sample with characteristics approximately representative of all U.S. children in this age range [21]. The ABCD study is currently ongoing, with data collection occurring across 22 study sites in the U.S. [20]. Further description of the ABCD study procedures can be found elsewhere [22]. Clearance was obtained from all relevant institutional review/research ethics boards and informed written consent and written assent were obtained from all caregivers and youth, respectively. After approval for use, data were accessed via the National Institute of Mental Health Data Archive (https://nda.nih.gov). We used data from ABCD Release 4.0 (released in September 2021), which contains full baseline (2017–2018) and one-year follow-up (2018–2019) data for the ABCD cohort, as these were the only longitudinal data available occurring entirely before the COVID-19 pandemic.

### Measures

#### Screen time

Youth self-reported their screen time via the ABCD Youth Screen Time Survey, which is based on a previously validated measure [23]. Participants reported their usual time spent (hours per day) in six different forms of screen time on weekdays and weekend days separately. The forms of screen time measured were viewing/streaming television shows or movies, watching/streaming videos (e.g., YouTube), playing video games, texting, video chatting, and using social networking sites. Response options for each included none, < 30 min (coded as 0.5 h), 1 h, 2 h, 3 h, and 4 + hours (coded as 4.8 h). The highest response category was open-ended, so it was coded to be equal to 1.2 times the upper limit, consistent with prior work [24]. Next, weekday and weekend day reports of each form of screen time were combined to generate the usual daily time spent in each form of screen time (hours); calculated as (screen time on weekdays*0.71) + (screen time on weekend days*0.29) [25]. Usual daily screen time (hours) was then converted to minutes by multiplying by 60. Lastly, we collapsed the six different forms of screen time measured into three distinct categories (minutes per day): “streaming” (viewing/streaming television shows or movies + watching/streaming videos [e.g., YouTube]), “gaming” (playing video games), and “socializing” (texting + video chatting + social networking sites); these three categories were kept separate given evidence that different forms of screen time may have unique associations with weight-related outcomes [11, 26].

#### Physical activity

Youth self-reported their physical activity via the Youth Risk Behavior Surveillance System item, “During the past 7 days, on how many days were you physically active for a total of at least 60 min per day? (Add up all the time you spent in any kind of physical activity that increased your heart rate and made you breathe hard at least some of the time).” This item is validated and is commonly used in epidemiologic studies of youth with report-based measures of physical activity, but may underestimate physical activity [27, 28]. Response options ranged from zero to seven days. The reported days per week were converted to a weekly average (minutes per day) by multiplying responses by 60 min and dividing by 7. For example, participants who reported 2 days per week of physical activity were coded as 17.1 min per day of physical activity ([2 days per week*60 min per day]/7 total days).

#### Sleep duration

Caregivers reported youth sleep duration with a single item from the Sleep Disturbance Scale for Children, which has been validated for use in youth [29, 30] and is moderately correlated with device-based sleep duration [31]. The item was “How many hours of sleep does your child get on most nights?” with the following closed response options: less than 5 h, 5–7 h, 7–8 h, 8–9 h, and 9–11 h [29]. The responses were converted to minutes per day by taking the midpoint of each response category and multiplying by 60 min. For example, a reported sleep duration of 9–11 h was coded as 600 min per day (10 h*60 min). For those who reported “less than 5 h,” the midpoint between 0 to 5 h (2.5 h) was used for the conversion to minutes per day of sleep.

##### BMI

At baseline and one-year follow-up, BMI (kg⋅m^−2^) was calculated based on height (cm) and weight (kg; measured in triplicate) measured by trained ABCD study staff. BMI was converted to *z*-scores using the Centers for Disease Control and Prevention 2000 Growth Chart SAS software, which generates metrics based on age and sex [32].

#### Covariates

A priori covariates were baseline participant age (continuous; years), caregiver-reported child race/ethnicity (categorical; non-Hispanic Asian, non-Hispanic Black, Hispanic, Other [including Mixed Race], and non-Hispanic White), and socioeconomic status operationalized as the income-to-needs ratio. The income-to-needs ratio was calculated by dividing baseline caregiver-reported household income by the 2017 federal poverty threshold (based on household size), with higher values indicating higher socioeconomic status [33]. The income-to-needs ratio was categorized as below the poverty threshold (≤ 0.99), low socioeconomic status (1.00–1.99), intermediate socioeconomic status (2.00–3.99), and high socioeconomic status (≥ 4.00), consistent with prior work [34]. A final income category (“not reported”) was created for those missing an income-to-needs ratio (*n* = 972) due to either combined annual household income, household size, or both not being reported. The abovementioned covariates were selected given prior evidence that age, race/ethnicity, and income are associated with behavior and BMI [35–38]. Lastly, continuous baseline internalizing (depressive, anxiety) symptom raw score (possible range: 0–64), based on caregiver reports via the Child Behavior Checklist [39], was included given associations between internalizing symptoms, screen time, and weight status [40–42].

### Statistical analysis

To account for the ABCD study sampling/design features (e.g., clustering of participants within study sites), a complex survey design-based approach was used for all analyses. The study site was specified as the cluster variable and sample weights were applied to approximate the American Community Survey, consistent with analytic recommendations [43]. All analyses were sex-stratified (based on caregiver-reported sex assigned at birth), given sex differences in behavior and body composition during this developmental period [10, 12, 35]. Descriptive statistics for continuous variables (mean [SE]) were calculated. Discrete variables were reported as unweighted sample size (n) and weighted percentage (%). Model formulation (below) was informed by a Directed Acyclic Graph (DAG) representing the hypothesized interrelationships between the variables examined in the current study (Supplemental Fig. 1). The DAG created is consistent with prior work [44] and was based on a causal inference perspective on the analysis of compositional data previously reported [45].

#### Isotemporal substitution analysis

Our primary analytical approach was isotemporal substitution to estimate the associations between replacing different forms of screen time, physical activity, and sleep with BMI. Although replacements of any time increment can be modeled, we selected 30-min substitutions, consistent with prior work and given its real-world applicability [18, 46]. Accordingly, the streaming, gaming, socializing, physical activity, and sleep variables were each divided by a constant of 30. In addition, a total time variable was created (by summing time spent in all behaviors reported in the current study). The total time variable in the models controls for time, allowing for a direct comparison between behaviors and their association with BMI *z*-score. To model the replacements, linear regressions with all baseline behaviors, *except* the behavior being replaced were run. The parameter estimates for each of the remaining behavioral predictors in the model represent the associations between behavioral replacements and BMI *z*-score. Models were systematically run dropping one behavior at a time so that each behavioral replacement combination was estimated. These analyses were conducted in SAS v. 9.4 using the SURVEY procedures.

#### Compositional data analysis

To examine these associations using an alternative analytic method, CoDA was conducted as a secondary analysis. Different data handling procedures were needed to conduct CoDA, which is also designed for data that make up portions of a finite whole (i.e., the 24-h day) [47–50]. Given the activity behavior survey items/response options used in the ABCD study, it was possible to report less than a full 24-h day. Thus, an “other activities” category was calculated as the remaining amount of time after time spent in screen time, physical activity, and sleep were summed. Because data are isometric logarithmic transformed, CoDA also requires positive non-zero values for every composition component. Zero minutes per day of physical activity (*n* = 574) was commonly reported, so we assigned 30 s of physical activity per day to those participants to maximize our analytic sample size; we maintained the relative proportions of the remaining behaviors using the multiplicative replacement method [51]. Sensitivity analyses without 30-s assignments for the physical activity variable yielded qualitatively similar results to those reported below (Supplemental Table 1). There were no circumstances where the “other activities” and sleep variables were assigned 30 s, as 0 min per day of “other activities” was not observed and the lowest possible value to report for sleep was 2.5 h per day (150 min per day; see Sleep Duration). Streaming, gaming, and socializing were not assigned 30 s in circumstances where 0 min per day was reported; this is because these were the main behaviors of interest and due to the high occurrence of 0 min per day of socializing reported (*n* = 4,623). Therefore, those with 0 min per day reported for any form of screen time were excluded from the CoDA analytic sample. Because of this, the CoDA analytic sample size was about half of the isotemporal substitution linear regression analytic sample size. We compared those included in the CoDA analytic sample (> 0 min per day of each form of screen time) to those excluded from the CoDA analytic sample by participant characteristics, behaviors, and BMI *z*-score using weighted independent samples t-tests and chi-square tests.

Analysis using the CoDA method involves two data transformations before statistical modeling. The first data transformation converts the individual composition component values (e.g., time spent in each behavior) to proportions of the total time. The proportion of each behavior was calculated by dividing each child’s amount of time in each behavior by 1,440 min. The second data transformation implements an isometric logarithmic ratio (ILR) transformation to remove multicollinearity, which allows for regression modelling of the transformed variables [47–49].

Compositional means (proportion of the day) and compositional variation matrices were calculated to describe variability of the transformed behavioral data. Compositional variation matrices are a series of log ratios of pair-wise variation of two behaviors to describe the covariance structure independently of data transformation [19]. Two behaviors that are perfectly proportional will have a covariance of zero, while higher covariance values indicate behaviors with lower proportionality. CoDA relies on sequential binary partitioning, which requires an ordering of the composition components [52]. While the ordering is arbitrary, standard practice is to place the component of interest in the first position. We conducted separate linear regressions with each baseline behavior rotated as the first composition component as the exposure and BMI *z*-score at follow-up as the outcome. Each model was adjusted for the set of a priori covariates mentioned above. Significance was set a priori as when 95% confidence intervals (95%CI) did not cross zero. These analyses were conducted in R using the *‘survey’* package [53, 54].

#### Data exclusions

**Fig. 1:**
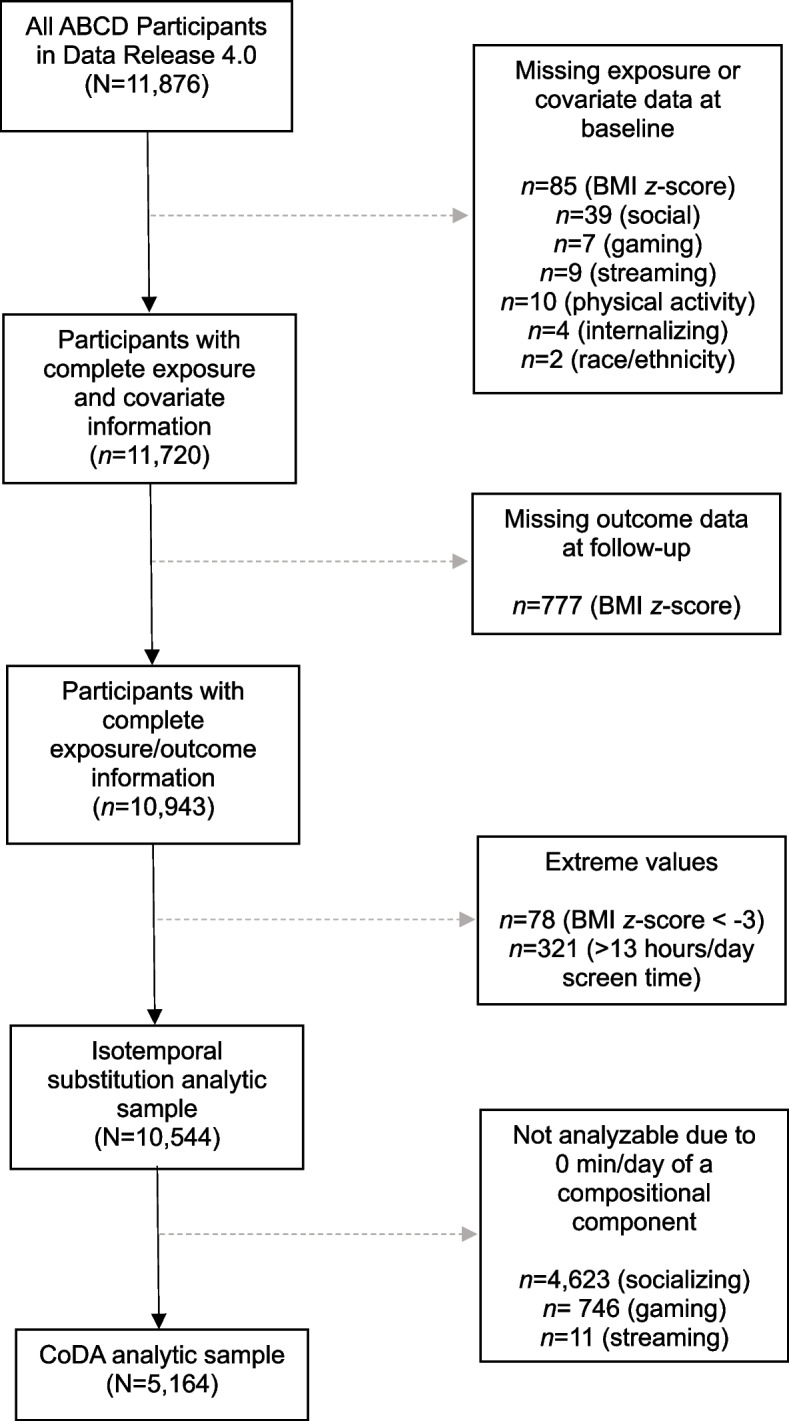
Study participant flow

Participants were excluded from the analytic sample if they had missing exposure (screen time, physical activity, and sleep duration), covariate, or outcome data at baseline, or missing outcome data at follow-up. In addition, participants with extremely low BMI *z*-scores (< -3.0) at baseline or follow-up were considered outliers and omitted from analyses because growth charts may not accurately track growth in these children [55]. Lastly, participants who simultaneously reported 1 h per day of physical activity, 10 h per day of sleep, and > 13 h per day of screen time at baseline were excluded because these values summed to > 24 h per day (resulting in a negative value for “other activities” in the CoDA models); sensitivity analyses with these participants included yielded qualitatively similar results to those reported below (data not shown). Figure 1 provides more details on participant exclusion flow.

## Results

### Isotemporal substitution analysis

Of the original 11,876 participants enrolled in the ABCD Study at baseline, 10,544 were included in the isotemporal substitution analytic sample. Many of these exclusions were due to missing BMI *z*-score at follow-up (*n* = 777). Of the analytic sample, 5,058 (48.9%) were female, and the baseline age was 9.9 (0.03) years. About half (45.4%) of the participants were non-White, with a wide range of socioeconomic status (income-to-needs ratio range: 0.1–15.4). Time spent in streaming was 139.9 (5.2) minutes per day, in gaming was 61.9 (2.3) minutes per day, and in socializing was 26.8 (1.9) minutes per day. Participants reported 29.5 (0.7) minutes per day of physical activity and 538.1 (3.4) minutes per day of sleep. The mean BMI *z*-score was 0.45 (0.05) at baseline and 0.52 (0.05) at follow-up. See Table 1 for these characteristics stratified by sex.

**Table 1:** Descriptive statistics in the isotemporal substitution analytic sample by sex (*N* = 10,544)

	**Females (*n* = 5,058)**	**Males (*n* = 5,486)**
Age, Years	9.9 (0.03)	10.0 (0.03)
Race/Ethnicity		
*Asian*	120 (4.0%)	107 (3.3%)
*Black*	693 (12.3%)	679 (11.0%)
*Hispanic*	996 (23.0%)	1,095 (23.5%)
*Other/Multiracial*	534 (6.9%)	565 (6.7%)
*White*	2,715 (53.8%)	3,040 (55.5%)
Income-to-Needs Ratio		
≤ *0.99*	621 (15.5%)	643 (15.1%)
*1.00–1.99*	713 (18.4%)	781 (18.9%)
*2.00–3.99*	1,171 (25.3%)	1,255 (25.3%)
≥ *4.00*	2,109 (30.7%)	2,279 (30.0%)
*Not Reported*	444 (10.1%)	528 (10.7%)
Internalizing Symptoms	5.2 (0.2)	5.2 (0.2)
Streaming, Min/Day	134.6 (5.2)	144.9 (5.3)
Gaming, Min/Day	40.2 (1.8)	82.7 (3.3)
Socializing, Min/Day	31.5 (2.0)	22.3 (1.9)
Physical Activity, Min/Day	28.6 (0.7)	30.3 (0.7)
Sleep, Min/Day	537.9 (3.4)	538.3 (3.6)
Baseline BMI-*z* Score	0.43 (0.05)	0.48 (0.06)
Follow-up BMI-*z* Score	0.49 (0.05)	0.55 (0.05)

Continuous variables are reported as weight mean (SE), and discrete variables are reported as n (weighted %)

Sex-stratified isotemporal substitution model estimates are presented in Table 2, which reflect the longitudinal association between 30-min behavioral replacements and BMI *z*-score one year later. In females, replacing 30 min of socializing (β [95% CI] = -0.03 [-0.05, -0.002]), streaming (-0.03 [-0.05, -0.01]), gaming (-0.03 [-0.06, -0.01]), or sleep (-0.02 [-0.05, -0.003]) with 30 min of physical activity was associated with a lower BMI *z*-score one year later. Thirty-minute reallocations between the different forms of screen time and sleep were not associated with BMI *z*-score one year later. Given the above model estimates, a female with a BMI *z*-score of 0.50 who reallocates 30 min of any form of screen time with 30 min of physical activity at baseline would be estimated to have a BMI z-score of 0.47 at one-year follow-up.

**Table 2:** Isotemporal substitution (30-min substitutions) between different forms of screen time, physical activity, and sleep at baseline predicting follow-up BMI *z*-score in females and males (β [95% CI]) (*N* = 10,544)

	**Decrease by 30 min**				
	**Females (*n* = 5,058)**				
**Increase by 30 min**	↓ Socializing	↓ Streaming	↓ Gaming	↓ Physical Activity	↓ Sleep
↑ Socializing	-	-0.003 (-0.01, 0.01)	-0.005 (-0.02, 0.01)	0.03 (0.002, 0.05)*	0.002 (-0.01, 0.01)
↑ Streaming	.003 (-0.01, 0.01)	-	-0.002 (-0.02, 0.01)	0.03 (0.01, 0.05)**	0.005 (-0.004, 0.01)
↑ Gaming	0.005 (-0.01, 0.02)	0.002 (-0.01, 0.02)	-	0.03 (0.01, 0.06)*	0.01 (-0.01, 0.02)
↑ Physical Activity	-0.03 (-0.05, -0.002)*	-0.03 (-0.05, -0.01)**	-0.03 (-0.06, -0.01)*	-	-0.02 (-0.05, -0.003)*
↑ Sleep	-0.002 (-0.01, 0.01)	-0.005 (-0.01, 0.004)	-0.01 (-0.02, 0.01)	0.02 (0.003, 0.05)*	-
	**Males (*n* = 5,486)**				
↑ Socializing	-	0.01 (-0.002, 0.02)	0.01 (0.0001, 0.02)*	0.004 (-0.03, 0.04)	0.03 (0.01, 0.05)**
↑ Streaming	-0.01 (-0.02, 0.002)	-	0.001 (-0.005, 0.01)	-0.01 (-0.04, 0.02)	0.02 (0.01, 0.03)***
↑ Gaming	-0.01 (-0.02, -0.0001)*	-0.001 (-0.01, 0.005)	-	-0.01 (-0.04, 0.02)	0.02 (0.01, 0.03)***
↑ Physical Activity	-0.004 (-0.04, 0.03)	0.01 (-0.02, 0.04)	0.01 (-0.02, 0.04)	-	0.02 (-0.01, 0.05)
↑ Sleep	-0.03 (-0.05, -0.01)**	-0.02 (-0.03, -0.01)***	-0.02 (-0.03, -0.01)***	-0.02 (-0.05, 0.01)	-

Models were adjusted for participant age, race/ethnicity, socioeconomic status, internalizing symptoms, and BMI *z*-score at baseline. Time spent in each behavior (except the behavior being replaced) and total time were simultaneous predictors in each model. *P < .05, **P < .01, ***P < .001

For males, replacing 30 min of socializing (β [95% CI] = -0.03 [-0.05, -0.01]), streaming (-0.02 [-0.03, -0.01]), or gaming (-0.02 [-0.03, -0.01]) with 30 min of sleep was associated with a lower BMI *z*-score one year later. Thirty-minute replacements between different forms of screen time and physical activity were not associated with BMI *z*-score one year later. Lastly, replacing 30 min of socializing with 30 min of gaming was associated with a lower BMI *z*-score one year later (-0.01 [-0.02, -0.0001]).

### Compositional data analysis

An additional 5,380 participants were excluded from the CoDA analytic sample due to having 0 min per day in one form of screen time, therefore 5,164 participants were analyzed with CoDA. Of this analytic sample, 50.4% were female, the baseline age was 10.0 (0.03) years, and just under half (47.7%) were non-White. See Table 3 for additional participant characteristics and for a comparison between those included (*n* = 5,164) and excluded (*n* = 5,380) from the CoDA analysis. Notably, there were differences between these samples by sex and race; there were fewer female and Black participants and more White participants excluded from the CoDA analysis compared with those included. Those excluded from the CoDA analysis reported less time in streaming, gaming, and socializing and they also had lower BMI *z*-scores at baseline and follow-up compared with those included in the CoDA analysis.

**Table 3:** Comparison between those included in the CoDA analytic sample and those excluded from the CoDA analytic sample (due to 0 min per day of any form of screen time)

	**Included** **(*n* = 5,164)**	**Excluded** **(*n* = 5,380)**	*P*
Age, Years	10.0 (0.03)	9.9 (0.02)	< .0001
Female	2,542 (50.4%)	2,516 (47.4%)	.0404
Race/Ethnicity			
*Asian*	110 (3.7%)	117 (3.7%)	
*Black*	827 (13.9%)	545 (9.3%)	
*Hispanic*	1,011 (22.9%)	1,080 (23.6%)	.0011
*Other/Multiracial*	549 (7.2%)	550 (6.3%)	
*White*	2,667 (52.3%)	3,088 (57.1%)	
Income-to-Needs Ratio			
≤ *0.99*	634 (15.1%)	630 (15.4%)	
*1.00–1.99*	756 (19.1%)	738 (18.2%)	
*2.00–3.99*	1,238 (26.4%)	1,188 (24.2%)	.0941
≥ *4.00*	2,041 (28.8%)	2,347 (32.0%)	
*Not reported*	495 (10.6%)	477 (10.2%)	
Internalizing Symptoms	5.1 (0.2)	5.3 (0.2)	.1272
Streaming (min/day)	151.9 (4.8)	127.5 (5.3)	< .0001
Gaming (min/day)	72.7 (2.1)	50.8 (2.2)	< .0001
Socializing (min/day)	46.8 (2.0)	6.2 (0.7)	< .0001
Physical Activity (min/day)	29.7 (0.5)	29.2 (0.8)	.3753
Sleep (min/day)	534.9 (3.4)	541.4 (3.5)	.0006
Baseline BMI-*z* Score	0.50 (0.05)	0.41 (0.06)	.0067
Follow-up BMI-*z* Score	0.57 (0.05)	0.47 (0.05)	.0028

Continuous data presented as weighted mean (SE), discrete data presented as n (weighted %). *P*-values derived from weighted independent samples t-test or weighted chi-square tests. Those excluded from the CoDA analytic sample due to 0 min/day screen time were included in the isotemporal substitution analytic sample

The compositional means (proportions of the day) for time spent in each behavior stratified by sex are presented in Table 4. The proportion of time spent in each behavior was similar for females and males, except for the proportion of time spent in gaming (females: 2.6%; males: 4.6%) and other activities (females: 45.7%; males: 42.8%). The variability of the data stratified by sex is presented in the compositional variation matrix found in Table 5. The lowest covariance value observed was between sleep and other activities (females: 0.2; males: 0.2). The highest covariance values observed were between physical activity and each form of screen time for females (socializing: 15.0, streaming: 15.3, gaming: 15.3) and males (socializing: 13.0, streaming: 13.2, gaming: 13.6). In CoDA, lower covariance values reflect behaviors with higher proportionality, whereas higher covariance values reflect behaviors with lower proportionality.

**Table 4:** Compositional means (proportions of the day) of behaviors by sex (*n* = 5,164)

	**Females** **(*n* = 2,542)**	**Males** **(*n* = 2,622)**
Socializing	2.4%	2.1%
Streaming	8.1%	8.7%
Gaming	2.6%	4.6%
Physical Activity	0.5%	0.7%
Other Activities	45.7%	42.8%
Sleep Duration	40.8%	41.1%

The analytic sample does not contain participants with 0 min per day of socializing, streaming, or gaming

**Table 5:** Compositional variation matrix of pair-wise variation of two behaviors by sex

	**Socializing**	**Streaming**	**Gaming**	**Physical Activity**	**Other Activities**	**Sleep**
	**Females (*n* = 2,542)**					
Socializing	0.0					
Streaming	1.1	0.0				
Gaming	1.3	0.9	0.0			
Physical Activity	15.0	15.3	15.3	0.0		
Other Activities	1.3	1.2	1.4	14.0	0.0	
Sleep	0.9	0.7	0.9	13.9	0.2	0.0
	**Males (*n* = 2,622)**					
Socializing	0.0					
Streaming	1.1	0.0				
Gaming	1.2	0.9	0.0			
Physical Activity	13.0	13.2	13.6	0.0		
Other Activities	1.2	1.2	1.5	12.2	0.0	
Sleep	0.8	0.7	1.0	12.0	0.2	0.0

The values in the compositional variation matrix are log ratios of pair-wise variation of two behaviors. A zero value within the compositional variation matrix is indicative of the two behaviors being completely proportional and whereas higher values are indicative of the two behaviors having lower proportionality. The analytic sample does not contain participants with 0 min per day of socializing, streaming, or gaming

Table 6 presents the sex-stratified compositional model estimates of the association between the proportion of time spent in behavior at baseline and BMI *z*-score at follow-up. In males, a greater proportion of time spent in baseline socializing, at the expense of time spent equally in the remaining behaviors, was associated with a higher BMI *z*-score at follow-up (β [95% CI] = 0.05 [0.02, 0.08]). Therefore, a male with baseline BMI z-score of 0.50 who has a 1% (14.4 min/day) increase in socializing, relative to the remaining day at baseline, would be estimated to have a BMI z-score of 0.55 at follow-up. This association was not observed among females. No other statistically significant associations were observed between the baseline proportion of time spent in the other forms of screen time and follow-up BMI *z*-score. In males, a greater proportion of time spent in baseline physical activity, at the expense of time spent equally in the remaining behaviors, was associated with a higher BMI *z*-score one year later (0.01 [0.001, 0.01]). Further, in males, a greater proportion of time spent in baseline sleep, at the expense of time spent equally in the remaining behaviors, was associated with a lower BMI *z*-score one year later (-0.11 [-0.19, -0.04]). No statistically significant associations were observed in females.

**Table 6:** Compositional model estimates of the association (β[95% CI]) between baseline behavioral composition and follow-up BMI *z*-score by sex

	**Females (*n* = 2,542)**	**Males (*n* = 2,622)**
Socializing	-0.01 (-0.03, 0.01)	**0.05 (0.02, 0.08)**
Streaming	0.01 (-0.02, 0.04)	0.02 (-0.02, 0.05)
Gaming	0.01 (-0.02, 0.05)	0.01 (-0.01, 0.03)
Physical Activity	-0.004 (-0.01, 0.003)	**0.01 (0.001, 0.01)**
Sleep	-0.004 (-0.09, 0.08)	**-0.11 (-0.19, -0.04)**
Other Activities	-0.01 (-0.08, 0.07)	0.03 (-0.02, 0.09)

Models were adjusted for participant age, race/ethnicity, socioeconomic status, internalizing symptoms, and BMI *z*-score at baseline. We report the isometric logarithmic ratio (ILR) 1 of each behavior rotation (12 total models). The analytic sample does not contain participants with 0 min per day of socializing, streaming, or gaming. Bolded estimates are statistically significant as indicated by a confidence interval that does not overlap with 0

## Discussion

Few studies have examined the associations between BMI and contemporary aspects of time-use such as different forms of screen time (streaming, gaming, and socializing) along with traditional elements of time-use including physical activity and sleep. Our findings suggest that the one-year longitudinal associations between screen time and BMI in youth may depend on the form of screen time, what other behavior it replaces (physical activity or sleep), and participant sex. Results also differed by the statistical approach used to estimate the associations at hand. While the magnitude of the associations reported here are small and may not represent clinically meaningful associations with BMI across one year, our findings are important because of the pervasiveness of screen time worldwide [56] and potential for these associations to accumulate over time longer periods of time. It is also worth noting that we did not examine bi-directionality here; it is possible that that high BMI precedes unhealthy time-use behaviors [57–59], and future work examining the possibility of bi-directionality could increase our understanding of the associations at hand.

We extend prior knowledge from longitudinal studies of the association between screen time and BMI in the ABCD cohort by (1) examining the independent associations of different forms of screen time, (2) estimating replacement effects with physical activity and sleep, and (3) stratifying by sex. Prior work in this cohort that examined different forms of screen time separately in relation to BMI found that every form of screen time *except* social networking was related to higher BMI one year later [9]. This is not entirely consistent with what we report here, which could be due to a couple of reasons. First, we included all forms of screen time simultaneously in our models, whereas prior work did not [9]; therefore, our findings better account for the non-independence of time spent in different activities over the course of the day. Second, physical activity and sleep duration were not accounted for in this previous analysis, despite being important influences on BMI [9]; our results highlight that the association between screen time and BMI may depend on whether it displaces physical activity or sleep. A second study of the ABCD cohort that examined screen time, physical activity, and sleep simultaneously in relation to BMI and found that those who met any combination of screen time (≤ 2 h per day), sleep (9–11 h per night), or physical activity (60 min per day, 7 days per week) recommendations had lower BMI over time compared with those who did not meet any behavioral recommendations [60]. This work importantly demonstrated that a combination of behaviors may be more salient than a single behavior alone for weight outcomes; however, this study did not examine different forms of screen time as distinct behaviors [60]. In addition, neither of the abovementioned studies reported sex-specific associations [9, 60] and our results suggest that sex-stratification is important for increasing our understanding of the associations at hand.

Using isotemporal substitution modeling, we found that screen time was related to higher BMI when it was at the expense of physical activity in females, whereas screen time was related to higher BMI when it was at the expense of sleep in males. Around the onset of puberty, physical activity levels tend to decline, with females becoming less physically active than males [61]. Similarly, sleep duration declines from childhood through adolescence [62] and sex-related differences in sleep health also emerge around puberty [63]. Females tend to have longer sleep periods and poorer sleep quality compared with males [63]. In the current study, females and males had similar time spent in physical activity and sleep duration, indicating these data were collected prior to the onset of sex differences observed in past studies. Further sex-stratified investigation of the relationship between screen time, physical activity, sleep, and BMI as the ABCD cohort ages throughout adolescence will be informative for understanding whether the associations observed here are stable across development.

Past studies have suggested that it is especially important to promote physical activity in peri-pubertal females before the abovementioned maturation-related declines in physical activity occur [61]. Puberty is a period of rapid biopsychosocial change [64] and beyond increasing energy expenditure, physical activity offers psychosocial benefits that screen time may not, such as increasing body esteem and physical self-efficacy [65, 66], which can each further indirectly support healthy weight [67, 68]. Alternatively, our findings suggest that increasing sleep duration, specifically at the expense of screen may be more beneficial for male (compared with female) youth. It is possible that male youth are more likely to use screens in ways that interfere with sleep duration compared with female youth; males report spending more time watching television/videos and video gaming in bed compared with females [69]. It is also worth noting that other metrics of sleep, such as social jetlag, may be related to weight outcomes in males and females independent of sleep duration [70]. At the onset of puberty, there is a natural shift to later circadian preferences [71], meaning social jetlag increases around this developmental period [70]. Therefore, future research in peri-pubertal youth may need to consider addressing other sleep characteristics beyond what was examined in the current study.

We further observed that replacing 30 min of socializing with 30 min of gaming was associated with a lower BMI in males using isotemporal substitution. The CoDA analysis gave similar results in males; a greater proportion of time spent in socializing, relative to the remaining behaviors, was related to higher BMI. These results are consistent with prior work [72] and warrant further investigation of social screen time in relation to BMI in males. Other studies of social screen time and weight status have yielded inconsistent findings [73, 74]. A recent study of over 120,000 adolescents found that social media use was related to a greater risk of overweight/obesity, noting breakfast skipping, life satisfaction, and family communication as potential explanatory pathways [73]. Another study that used CoDA to generate sedentary behavioral compositions (comprised of 15 different types of sedentary behaviors) reported that individuals with overweight/obesity spent a greater proportion of time in socially disengaged sedentary behaviors compared with those with healthy weight [74]. Different forms of screen time, including social screen time, should be further examined in relation to weight outcomes using isotemporal substitution and CoDA when possible.

In the current study, the isotemporal substitution and CoDA statistical approaches yielded rather different results. The most notable differences in findings between the two approaches were among females; isotemporal substitution identified significant associations between behavior and BMI while CoDA did not. There are at least two reasons why the results of these statistical approaches differed: 1) they were based on very different analytic samples and 2) untransformed data were used in isotemporal substitution models, while transformed data were used in CoDA. Consequently, they address the research question in different ways; isotemporal substitution analyses estimated linear behavioral replacements in relation to BMI while CoDA estimated proportions of behaviors in relation to BMI in the present study [17, 19]. Therefore, the coefficients from each set of models have different interpretations and are not necessarily directly comparable. Nevertheless, we believe presenting both sets of results from these two statistical methods is a strength of the current work, as there is no definitive way to determine which approach is most appropriate. Complementary results may increase confidence in the findings and differing results highlight the need for further follow-up in the ABCD cohort and additional study designs addressing the effects of screen time on BMI. As causality cannot be determined with the current observational work, experimental studies manipulating screen time could strengthen the limited evidence to date for causal associations between screen time and obesity.

There are some limitations to the present study. First, dietary intake information was not collected in the ABCD Study. While dietary intake likely explains some of the association between screen time and BMI, prior evidence suggests that screen time is related to adiposity even after accounting for dietary intake [26, 75]. Nonetheless, future research could benefit from identifying cohorts that examine both time-use and eating behavior, such as snacking for further analysis. Second, we did not adjust for pubertal status due to the large number of missing observations for this variable, consistent with previous reports in the ABCD cohort [42, 76]. Prior studies suggest that baseline age performs similarly to baseline pubertal status as a covariate, likely due to a majority of the ABCD participants being at pre- or early- pubertal stages at baseline [42, 76]. However, future research should consider adjusting for this variable as the cohort ages and a wider range of pubertal statuses are observed. Third, the high prevalence of zero minutes per day of the socializing variable could be considered a limitation; such zero values preclude inclusion in CoDA because of the use of logarithmic ratio transformation. Zero values can be replaced with small time intervals [51], but we did not feel this was appropriate given almost half the sample had zero values for socializing. As the ABCD cohort ages and social screen time becomes more prevalent, future work using CoDA will likely include the participants we were unable to analyze here.

There are also measurement-related limitations. First, the current study relied on self-reported or caregiver-reported behaviors, which are subject to reporting errors and biases [77]. While the physical activity item we used is common in epidemiological studies, the item wording likely did not capture time spent in light physical activity (e.g., walking). Similarly, the amount of time spent in moderate-to-vigorous physical activity was almost certainly underreported because the item specified “for at least 60 min per day”; the absence of a detailed time component in the survey item and the inclusion of only activities lasting at least 60 min therefore may not comprehensively reflect participants’ physical activity levels. Longitudinal device-based (FitBit) activity data will become available in a subset of ABCD participants in upcoming data releases. Future work could consider using these data to capture more nuanced aspects of physical activity. In addition, screen time is not necessarily sedentary; however, prior work has reported that screen time is associated with accelerometer-measured sedentary time [78]. Relatedly, not all sedentary time is spent on screens [79]. The surveys used in the ABCD Study did not capture non-screen sedentary behaviors (e.g., reading, homework). This is particularly relevant for the CoDA models presented; likely, the “other activities” category we created for this analysis represents a combination of primarily light physical activity and sedentary behavior. Lastly, in the interpretation of our results, we did not account for the possibility of simultaneous screen use, and beyond time-use, we did not examine the context or the quality of screen time. Future work could use a combination of device-based measures of activity, inactivity, and self-reported screen time including duration, type, context, and content to further understand their associations with health outcomes.

Strengths of this study are the use of a nationally (U.S) representative sample and prospective study design. Additional notable strengths include the examination of different forms of screen time separately, while simultaneously accounting for physical activity and sleep duration, and sex-stratification.

## Conclusions

We used isotemporal substitution modeling and CoDA to examine the combined associations between different forms of screen time, physical activity, sleep duration, and BMI one year later in the ABCD Study. We found screen time is associated with higher BMI, but this depends on what behavior it replaces (physical activity or sleep) and participant sex. We also provide evidence that socializing screen time may be specifically related to higher BMI in males, relative to the remaining behaviors. Further investigation of the observed associations here will be important for informing future behavioral interventions aimed at promoting a healthy weight in youth.

### Supplementary Information

**Supplementary Material 1. **

## Data Availability

The data analyzed in the current study are publicly accessible via the NIMH data archive (NDA; https://nda.nih.gov).

## Abbreviations

ABCD: Adolescent Brain Cognitive Development
BMI: Body mass index
CoDA: Compositional data analysis
DAG: Directed Acyclic Graph
ILR: Isometric logarithmic ratio
U.S.: United States

## References

[1] Stierman B, Afful J, Carroll MD, Chen T-C, Davy O, Fink S (2021). National health and nutrition examination survey 2017–March 2020 prepandemic data files development of files and prevalence estimates for selected health outcomes.

[2] Rundle AG, Factor-Litvak P, Suglia SF, Susser ES, Kezios KL, Lovasi GS (2020). Tracking of obesity in childhood into adulthood: effects on body mass index and fat mass index at age 50. Child Obes.

[3] Avgerinos KI, Spyrou N, Mantzoros CS, Dalamaga M (2019). Obesity and cancer risk: Emerging biological mechanisms and perspectives. Metabolism..

[4] Piché M-E, Tchernof A, Després J-P (2020). Obesity phenotypes, diabetes, and cardiovascular diseases. Circ Res.

[5] Liang Y, Hou D, Zhao X, Wang L, Hu Y, Liu J (2015). Childhood obesity affects adult metabolic syndrome and diabetes. Endocrine.

[6] Wiklund P, Törmäkangas T, Shi Y, Wu N, Vainionpää A, Alen M (2017). Normal-weight obesity and cardiometabolic risk: A 7-year longitudinal study in girls from prepuberty to early adulthood. Obesity.

[7] Llewellyn A, Simmonds M, Owen CG, Woolacott N (2016). Childhood obesity as a predictor of morbidity in adulthood: a systematic review and meta-analysis. Obes Rev.

[8] Nagata JM, Chu J, Ganson KT, Murray SB, Iyer P, Gabriel KP (2023). Contemporary screen time modalities and disruptive behavior disorders in children: a prospective cohort study. J Child Psychol Psychiatry.

[9] Nagata JM, Iyer P, Chu J, Baker FC, Gabriel KP, Garber AK (2021). Contemporary screen time usage among children 9–10-years-old is associated with higher body mass index percentile at 1-year follow-up: A prospective cohort study. Pediatr Obes.

[10] Nagata JM, Cortez CA, Cattle CJ, Ganson KT, Iyer P, Bibbins-Domingo K (2022). Screen time use among US adolescents during the COVID-19 pandemic: findings from the Adolescent Brain Cognitive Development (ABCD) study. JAMA Pediatr.

[11] Fang K, Mu M, Liu K, He Y (2019). Screen time and childhood overweight/obesity: A systematic review and meta-analysis. Child Care Health Dev..

[12] Stierman B, Ogden CL, Yanovski JA, Martin CB, Sarafrazi N, Hales CM (2021). Changes in adiposity among children and adolescents in the United States, 1999–2006 to 2011–2018. Am J Clin Nutr..

[13] Wu Y, Gong Q, Zou Z, Li H, Zhang X (2017). Short sleep duration and obesity among children: A systematic review and meta-analysis of prospective studies. Obes Res Clin Pract.

[14] Hills AP, Andersen LB, Byrne NM (2011). Physical activity and obesity in children. Br J Sports Med.

[15] Fomby P, Goode JA, Truong-Vu K-P, Mollborn S (2021). Adolescent technology, sleep, and physical activity time in two US cohorts. Youth Society.

[16] Cabré-Riera A, Torrent M, Donaire-Gonzalez D, Vrijheid M, Cardis E, Guxens M (2019). Telecommunication devices use, screen time and sleep in adolescents. Environ Res.

[17] Mekary RA, Willett WC, Hu FB, Ding EL (2009). Isotemporal substitution paradigm for physical activity epidemiology and weight change. Am J Epidemiol.

[18] Huang WY, Wong S, He G, Salmon J (2016). Isotemporal substitution analysis for sedentary behavior and body mass index. Med Sci Sports Exerc.

[19] Aitchison J (1982). The statistical analysis of compositional data. J Roy Stat Soc Ser B (Methodol).

[20] Auchter AM, Mejia MH, Heyser CJ, Shilling PD, Jernigan TL, Brown SA (2018). A description of the ABCD organizational structure and communication framework. Dev Cogn Neurosci.

[21] Garavan H, Bartsch H, Conway K, Decastro A, Goldstein R, Heeringa S (2018). Recruiting the ABCD sample: Design considerations and procedures. Dev Cogn Neurosci.

[22] Barch DM, Albaugh MD, Avenevoli S, Chang L, Clark DB, Glantz MD (2018). Demographic, physical and mental health assessments in the adolescent brain and cognitive development study: Rationale and description. Dev Cogn Neurosci.

[23] Sharif I, Wills TA, Sargent JD (2010). Effect of visual media use on school performance: a prospective study. J Adolesc Health.

[24] Amadou A, Ferrari P, Muwonge R, Moskal A, Biessy C, Romieu I (2013). Overweight, obesity and risk of premenopausal breast cancer according to ethnicity: a systematic review and dose-response meta-analysis. Obes Rev.

[25] Foweather L, Knowles Z, Ridgers ND, O’Dwyer MV, Foulkes JD, Stratton G (2015). Fundamental movement skills in relation to weekday and weekend physical activity in preschool children. J Sci Med Sport.

[26] Zink J, Liu B, Yang CH, Herrick KA, Berrigan D. Differential associations between television viewing, computer use, and adiposity by age, gender, and race/ethnicity in United States youth: A cross‐sectional NHANES analysis. Pediatric Obes. 2023;18(10):e13070. 10.1111/ijpo.13070 37580912

[27] Saint-Maurice PF, Sousa S, Welk G, Matthews CE, Berrigan D (2020). Report-based measures of physical activity: Features, considerations, and resources.

[28] Troped PJ, Wiecha JL, Fragala MS, Matthews CE, Finkelstein DM, Kim J (2007). Reliability and validity of YRBS physical activity items among middle school students. Med Sci Sports Exerc.

[29] Bruni O, Ottaviano S, Guidetti V, Romoli M, Innocenzi M, Cortesi F (1996). The Sleep Disturbance Scale for Children (SDSC) Construct ion and validation of an instrument to evaluate sleep disturbances in childhood and adolescence. J Sleep Res.

[30] Herwanto H, Lestari H, Warouw SM, Salendu PM (2018). Sleep disturbance scale for children as a diagnostic tool for sleep disorders in adolescents. Paediatr Indones.

[31] Mummaneni A, Kardan O, Stier AJ, Chamberlain TA, Chao AF, Berman MG (2023). Functional brain connectivity predicts sleep duration in youth and adults. Hum Brain Mapp.

[32] A SAS Program for the 2000 CDC Growth Charts (Ages 0 to <20 Years) 2016. Available from: https://www.cdc.gov/nccdphp/dnpao/growthcharts/resources/sas.htm. Accessed 15 Jan 2023.

[33] Gonzalez MR, Palmer CE, Uban KA, Jernigan TL, Thompson WK, Sowell ER (2020). Positive economic, psychosocial, and physiological ecologies predict brain structure and cognitive performance in 9–10-year-old children. Front Hum Neurosci.

[34] White SF, Voss JL, Chiang JJ, Wang L, McLaughlin KA, Miller GE (2019). Exposure to violence and low family income are associated with heightened amygdala responsiveness to threat among adolescents. Dev Cogn Neurosci.

[35] Belcher BR, Berrigan D, Dodd KW, Emken BA, Chou C-P, Spuijt-Metz D (2010). Physical activity in US youth: impact of race/ethnicity, age, gender, & weight status. Med Sci Sports Exerc.

[36] Anderson SE, Economos CD, Must A (2008). Active play and screen time in US children aged 4 to 11 years in relation to sociodemographic and weight status characteristics: a nationally representative cross-sectional analysis. BMC Public Health.

[37] Giddens NT, Juneau P, Manza P, Wiers CE, Volkow ND (2022). Disparities in sleep duration among American children: effects of race and ethnicity, income, age, and sex. Proc Natl Acad Sci.

[38] Ogden CL, Fryar CD, Hales CM, Carroll MD, Aoki Y, Freedman DS (2018). Differences in obesity prevalence by demographics and urbanization in US children and adolescents, 2013–2016. JAMA.

[39] Achenbach TM, Ruffle TM (2000). The Child Behavior Checklist and related forms for assessing behavioral/emotional problems and competencies. Pediatr Rev.

[40] Sampasa-Kanyinga H, Colman I, Goldfield GS, Janssen I, Wang J, Tremblay MS (2021). 24-hour movement behaviors and internalizing and externalizing behaviors among youth. J Adolesc Health.

[41] Castillo F, Francis L, Wylie-Rosett J, Isasi CR (2014). Depressive symptoms are associated with excess weight and unhealthier lifestyle behaviors in urban adolescents. Childhood obesity (Print).

[42] Zink J, O'Connor SG, Blachman-Demner DR, Wolff-Hughes DL, Berrigan D (2024). Examining the Bidirectional Associations Between Sleep Duration, Screen Time, and Internalizing Symptoms in the ABCD Study. J Adolesc Health..

[43] Heeringa SG, Berglund PA. A guide for population-based analysis of the Adolescent Brain Cognitive Development (ABCD) Study baseline data. BioRxiv. 2020;2020–02.

[44] Cabanas-Sánchez V, Esteban-Cornejo I, García-Esquinas E, Ortolá R, Ara I, Rodríguez-Gómez I (2021). Cross-sectional and prospective associations of sleep, sedentary and active behaviors with mental health in older people: a compositional data analysis from the Seniors-ENRICA-2 study. Int J Behav Nutr Phys Act.

[45] Arnold KF, Berrie L, Tennant PW, Gilthorpe MS (2020). A causal inference perspective on the analysis of compositional data. Int J Epidemiol.

[46] McAlister KL, Zink J, Chu D, Belcher BR, Dunton GF (2021). Cross-Sectional and Longitudinal Associations between Non-School Time Physical Activity, Sedentary Time, and Adiposity among Boys and Girls: An Isotemporal Substitution Approach. Int J Environ Res Public Health.

[47] Carson V, Tremblay MS, Chaput JP, Chastin SF (2016). Associations between sleep duration, sedentary time, physical activity, and health indicators among Canadian children and youth using compositional analyses. Appl Physiol Nutr Metab..

[48] Chastin SF, Palarea-Albaladejo J, Dontje ML, Skelton DA (2015). Combined Effects of Time Spent in Physical Activity, Sedentary Behaviors and Sleep on Obesity and Cardio-Metabolic Health Markers: A Novel Compositional Data Analysis Approach. PLoS ONE.

[49] Grgic J, Dorothea D, Bengoechea EG, Shrestha N, Bauman A, Olds T, Pedisic Z (2018). Health outcomes associated with reallocations of time between sleep, sedentary behaviour, and physical activity: a systematic scoping review of isotemporal substitution studies. Int J Behav Nutr Phys Act..

[50] Booker R, Holmes ME, Newton RL, Norris KC, Thorpe RJ, Carnethon MR (2022). Compositional Analysis of Movement Behaviors’ Association on High-Sensitivity C-Reactive Protein: The Jackson Heart Study. Ann Epidemiol.

[51] Rasmussen CL, Palarea-Albaladejo J, Johansson MS, Crowley P, Stevens ML, Gupta N (2020). Zero problems with compositional data of physical behaviors: a comparison of three zero replacement methods. Int J Behav Nutr Phys Act.

[52] Dumuid D, Pedisic Z, Stanford TE, Martin-Fernandez JA, Hron K, Maher CA (2019). The compositional isotemporal substitution model: A method for estimating changes in a health outcome for reallocation of time between sleep, physical activity and sedentary behaviour. Stat Methods Med Res.

[53] Walmsley R, Chan S, Smith-Byrne K, Ramakrishnan R, Woodward M, Rahimi K (2021). Reallocation of time between device-measured movement behaviours and risk of incident cardiovascular disease. Br J Sports Med..

[54] Lumley T (2020). Survey: Analysis of complex survey samples. R package version 4.0.

[55] Chambers M, Tanamas SK, Clark EJ, Dunnigan DL, Kapadia CR, Hanson RL, et al. Growth tracking in severely obese or underweight children. Pediatrics. 2017;140(6):e20172248. 10.1542/peds.2017-2248 PMC5703793 29114063

[56] Thomas G, Bennie JA, De Cocker K, Castro O, Biddle SJ (2020). A descriptive epidemiology of screen-based devices by children and adolescents: a scoping review of 130 surveillance studies since 2000. Child Indic Res.

[57] Skrede T, Aadland E, Anderssen SA, Resaland GK, Ekelund U (2021). Bi-directional prospective associations between sedentary time, physical activity and adiposity in 10-year old Norwegian children. J Sports Sci.

[58] Cairney J, Veldhuizen S (2017). Organized sport and physical activity participation and body mass index in children and youth: A longitudinal study. Prev Med Rep.

[59] Sokol RL, Grummon AH, Lytle LA (2020). Sleep duration and body mass: direction of the associations from adolescence to young adulthood. Int J Obes.

[60] Fung H, Yeo BT, Chen C, Lo JC, Chee MW, Ong JL (2023). Adherence to 24-Hour Movement Recommendations and Health Indicators in Early Adolescence: Cross-Sectional and Longitudinal Associations in the Adolescent Brain Cognitive Development Study. J Adolesc Health.

[61] Metcalf BS, Hosking J, Jeffery AN, Henley WE, Wilkin T (2015). Exploring the adolescent fall in physical activity: a 10-yr cohort study (EarlyBird 41).

[62] Williams JA, Zimmerman FJ, Bell JF (2013). Norms and trends of sleep time among US children and adolescents. JAMA Pediatr.

[63] Meers J, Stout-Aguilar J, Nowakowski S. Sex differences in sleep health. Sleep Health. 2019:21–9.

[64] Dorn LD, Hostinar CE, Susman EJ, Pervanidou P (2019). Conceptualizing puberty as a window of opportunity for impacting health and well-being across the life span. J Res Adolesc.

[65] Spruit A, Assink M, van Vugt E, van der Put C, Stams GJ (2016). The effects of physical activity interventions on psychosocial outcomes in adolescents: A meta-analytic review. Clin Psychol Rev.

[66] Lubans D, Richards J, Hillman C, Faulkner G, Beauchamp M, Nilsson M, et al. Physical activity for cognitive and mental health in youth: a systematic review of mechanisms. Pediatrics. 2016;138(3):e20161642. 10.1542/peds.2016-1642 27542849

[67] Carissimi A, Adan A, Tonetti L, Fabbri M, Hidalgo MP, Levandovski R (2017). Physical self-efficacy is associated to body mass index in schoolchildren. Jornal de Pediatria.

[68] Kamody RC, Thurston IB, Decker KM, Kaufman CC, Sonneville KR, Richmond TK (2018). Relating shape/weight based self-esteem, depression, and anxiety with weight and perceived physical health among young adults. Body Image.

[69] Lemola S, Perkinson-Gloor N, Brand S, Dewald-Kaufmann JF, Grob A (2015). Adolescents’ electronic media use at night, sleep disturbance, and depressive symptoms in the smartphone age. J Youth Adolesc.

[70] Malone SK, Zemel B, Compher C, Souders M, Chittams J, Thompson AL (2016). Social jet lag, chronotype and body mass index in 14–17-year-old adolescents. Chronobiol Int.

[71] Carskadon MA, Vieira C, Acebo C (1993). Association between puberty and delayed phase preference. Sleep.

[72] Sampasa-Kanyinga H, Colman I, Goldfield GS, Hamilton HA, Chaput J-P (2020). Sex differences in the relationship between social media use, short sleep duration, and body mass index among adolescents. Sleep Health.

[73] Oduro MS, Katey D, Morgan AK, Peprah P. Problematic social media use and overweight/obesity: explanatory pathway analysis of 124 667 in‐school adolescents in 39 high‐income countries. Pediatr Obes. 2023;18(11):e13073. 10.1111/ijpo.13073 37691184

[74] Compernolle S, Van Dyck D, De Cocker K, Palarea-Albaladejo J, De Bourdeaudhuij I, Cardon G (2018). Differences in context-specific sedentary behaviors according to weight status in adolescents, adults and seniors: a compositional data analysis. Int J Environ Res Public Health.

[75] Fletcher E, Leech R, McNaughton SA, Dunstan DW, Lacy K, Salmon J (2015). Is the relationship between sedentary behaviour and cardiometabolic health in adolescents independent of dietary intake?. A systematic review obesity reviews.

[76] Goldstone A, Javitz HS, Claudatos SA, Buysse DJ, Hasler BP, de Zambotti M (2020). Sleep disturbance predicts depression symptoms in early adolescence: initial findings from the adolescent brain cognitive development study. J Adolesc Health.

[77] Matthews CE, Moore SC, George SM, Sampson J, Bowles HR (2012). Improving self-reports of active and sedentary behaviors in large epidemiologic studies. Exerc Sport Sci Rev.

[78] Zink J, Belcher BR, Eldin Dzubur WK, O'Connor S, Huh J, Lopez N, et al. Association between self-reported and objective activity levels by demographic factors: ecological momentary assessment study in children. JMIR mHealth and uHealth. 2018;6(6):e150. 10.2196/mhealth.9592 PMC6043732 29954723

[79] Hoffmann B, Kobel S, Wartha O, Kettner S, Dreyhaupt J, Steinacker JM (2019). High sedentary time in children is not only due to screen media use: A cross-sectional study. BMC Pediatr.

