# Supplementary material for: Longitudinal associations of screen time, physical activity, and sleep duration with body mass index in U.S. youth
Source: Int J Behav Nutr Phys Act. 2024 Apr 2;21:35. doi: 10.1186/s12966-024-01587-6 (PMC10988901; doi:10.1186/s12966-024-01587-6)
Supplement: Supplementary file 1 — Supplementary Material 1. [file 12966_2024_1587_MOESM1_ESM.docx]

**Supplemental Figure 1.** Directed Acyclic Graph depicting the hypothesized interrelationships between variables examined in the current study.


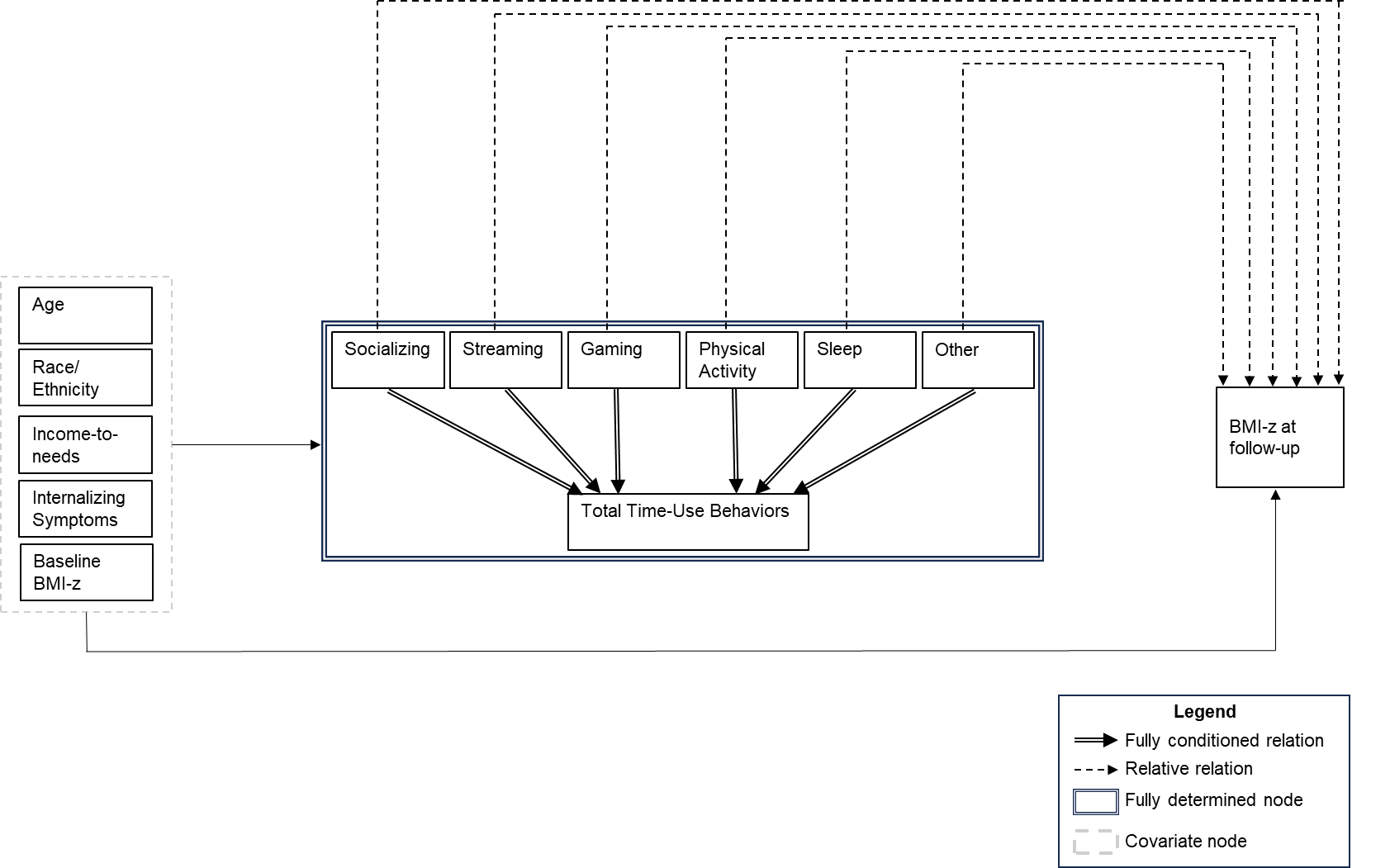


**Supplemental Table 1.** Compositional model estimates of the association (β[95% CI]) between baseline behavioral composition and follow-up BMI *z*-score by sex without 30-second assignments for PA (N=4,590).

|  |  | **Females (*n*=2,233)** | **Males (*n*=2,357)** |
| --- | --- | --- | --- |
|  | Socializing | -0.01 (-0.02, 0.02) | **0.06 (0.02, 0.09)** |
|  | Streaming | 0.01 (-0.02, 0.05) | 0.01 (-0.03, 0.05) |
|  | Gaming | 0.01 (-0.02, 0.05) | 0.01 (-0.01, 0.04) |
|  | Physical Activity | -0.04 (-0.08, 0.01) | 0.002 (-0.04, 0.04) |
|  | Sleep | 0.01 (-0.10, 0.12) | **-0.13 (-0.23, -0.03)** |
|  | Other Activities | 0.01 (-0.07, 0.09) | 0.04 (-0.02, 0.10) |

*Note.* Models were adjusted for participant age, race/ethnicity, socioeconomic status, internalizing symptoms, and BMI *z*-score at baseline. We report the isometric logarithmic ratio (ILR) 1 of each behavior rotation (12 total models). The analytic sample does not contain participants with 0 minutes per day of physical activity, socializing, streaming, or gaming. Bolded estimates are statistically significant as indicated by a confidence interval that does not overlap with 0.
